# Mechanism of Alzheimer's disease treatment by sound and light stimulation

**DOI:** 10.1016/j.apsb.2025.03.031

**Published:** 2025-03-15

**Authors:** Lixuan Ren, Xiwen Ma, Jianping Ye

**Affiliations:** a Institute of Trauma and Metabolism, Zhengzhou Central Hospital Affiliated to Zhengzhou University, Zhengzhou 450007, China; b Tianjian Laboratory of Advanced Biomedical Sciences, Academy of Medical Sciences, Zhengzhou University, Zhengzhou 450001, China

**Keywords:** A*β*, Multisensory stimulation, Lymphatic drainage, Alzheimer's disease

The brain lymphatic system has been a promising target in the treatment of Alzheimer's disease (AD)^1^. Induction of the lymphatic system function is an ideal approach in the development of new therapy for AD. A study published in *Nature* in 2024 introduced an innovative non-invasive approach in the promotion of the lymphatic function, which uses visual and auditory stimulation at specific frequencies to modulate neuron activity, inducing the brain lymphatic function for clearance of Amyloid-*β* (A*β*) from the neurons' microenvironment of brain^2^.

Millions of neurons are actively firing in the brain every day, organizing the body's movements, thoughts, and behaviors. The brain activity generates a large volume of metabolic wastes. Neurons coordinate their actions to promote removal of the wastes through large-amplitude, rhythmic ionic oscillations in the interstitial fluid (ISF) during sleep or ketamine anesthesia. The ionic waves facilitate the waste clearance by pushing fresh cerebrospinal fluid (CSF) through the parenchyma into the lymphatic system^3^. The brain lymphatic system contains four major parts^4^: the glymphatic system, the meningeal lymphatic vessels, the nasopharyngeal lymphatic plexus and the deep cervical lymphatic system. The glymphatic system, formed by the brain glial cells, is the starting point of the brain lymphatic system acting through the exchange of CSF and ISF to bring away the metabolic wastes^5^. Then, the metabolic wastes enter the meningeal lymphatic vessels through the exchange of CSF and lymph fluid to remove largemolecule wastes including A*β*, a protein that accumulates in the microenvironment of AD patients' brains as a major pathological feature^6^. The meningeal lymphatic vessels lead the waste-loaded lymph fluid to the nasopharyngeal lymphatic plexus and drain into the deep cervical lymphatic system eventually. With age, the function efficiency of the lymphatic system decreases, leading to the accumulation of A*β* in the microenvironment.

Current methods for non-invasive brain stimulation include light stimulation, sound stimulation, electrical stimulation and mechanical stimulation. Mechanical stimulation such as 40 Hz transcranial vibration stimulation (TVS) is effective to enhance spontaneous brain activity, synchronize overall brain activities through improving the lymphatic clearance function of brain^7^. Sound is also a powerful and non-invasive method to stimulate certain neuron activities in the brain. A study suggests that using sound to induce electric waves of brain may help people with dementia or cognitive decline to make them sleep better^8^. The alpha oscillation, approximately 10 Hz, is a defining electrophysiological feature of the conscious brain in human. Alpha oscillations have been associated with fundamental processes including memory and perception^8^. It is believed that neuronal excitability varies as a function of alpha phase of oscillation, since neurons are more active in firing during alpha oscillations, and during periods of lower alpha amplitude^9^. Researchers have applied an innovative brain modulation technique called Alpha Closed-Loop Auditory Stimulation (*α*CLAS), selectively adjusting human alpha oscillations and sleep. In the future, it may be possible to explore certain methods to counteract the observed decline in alpha frequency in the elderly and those with cognitive decline/dementia, thereby enhancing cognition and sleep, and ultimately benefiting patients with dementia^8^. Light treatment has become another focal point in the development of AD treatments. Zhan Yang's team used optogenetic methods to stimulate microglia *in vitro* and *in vivo*, leading to changes in microglia morphology that enhanced phagocytosis, effectively promoting the clearance of A*β* in the brain parenchyma^10^. Also, a non-invasive transcranial light treatment with a laser wavelength of 808 nm was performed on elderly mice and AD mice by Feifan Zhou's team^11^. Light treatment promoted meningeal lymphatic vessels (mLVs) expansion and drainage by restoring mitochondrial homeostasis in meningeal lymphatic endothelial cells (mLECs), eventually improving the cognitive abilities of AD mice. These studies show that both sound and light stimulation can serve as new approaches for treating neurodegenerative diseases, with varying degrees of improvement in cognitive dysfunction. What would occur if these two methods—sound and light—were combined?

Tsai's group answered this question in a recent study published in *Nature* (Fig. 1)^2^. They found that multisensory stimulation, combining sound and light, can reduce A*β* burden across the entire cortex in mice. Multisensory gamma wave stimulation is an emerging non-invasive neuromodulation technology that activates neural activity in specific brain regions by simultaneously stimulating multiple sensory systems, such as vision and hearing, to induce gamma (*γ*) wave of brain's electrophysiological activity. *γ* waves, a type of high-frequency neural oscillatory activity in the brain with a frequency range of 30–80 Hz, are believed to be associated with various cognitive functions, including attention, memory, and perceptual synchronization^2^. The researchers placed mice in chambers individually for multisensory stimulation. Each chamber was illuminated by a light-emitting diode programmed to either 8 Hz (125 ms light on, 125 ms light off), 40 Hz (12.5 ms light on, 12.5 ms light off, 60 W) or 80 Hz. Speakers were placed above the chambers and programmed to present a 10 kHz tone for 1 ms in duration and delivered at 60 decibels tones at 8 Hz or tones at 40 Hz. The researchers applied different frequencies of multisensory gamma stimulation to 6-month-old 5 × FAD mice (a model of AD). The results showed that mice receiving 40 Hz stimulation for 1 h had a significant reduction in A*β* levels in their brains compared to the control group (*n* = 10 (no stimulation), *n* = 5 (8 Hz), *n* = 8 (40 Hz), and *n* = 4 (80 Hz) in 6-month-old 5×FAD mice; *P-values* were analyzed by one-way ANOVA and Dunnett's multiple comparisons test), especially in the hippocampal region and cortex. By monitoring the dynamics of CSF, they demonstrated that multisensory 40 Hz stimulation mediated the clearance of A*β* through the glymphatic system. Compared to the control group, mice receiving the multisensory gamma stimulation showed increased clearance rate of ISF and an increased amount of CSF in brain. Additionally, the diameter of lymphatic vessels draining fluid in the neck region was observed to increase, and A*β* was accumulated in cervical lymph nodes. Aquaporin-4 (AQP4) water channels in astrocytes were shown to play a role in glymphatic transport^2^. By inhibiting and reducing AQP4, the authors found that cognitive performance was affected and the A*β* burden increased, suggesting that multisensory 40Hz stimulation promotes AQP4-dependent clearance of A*β*.

**Figure 1:**
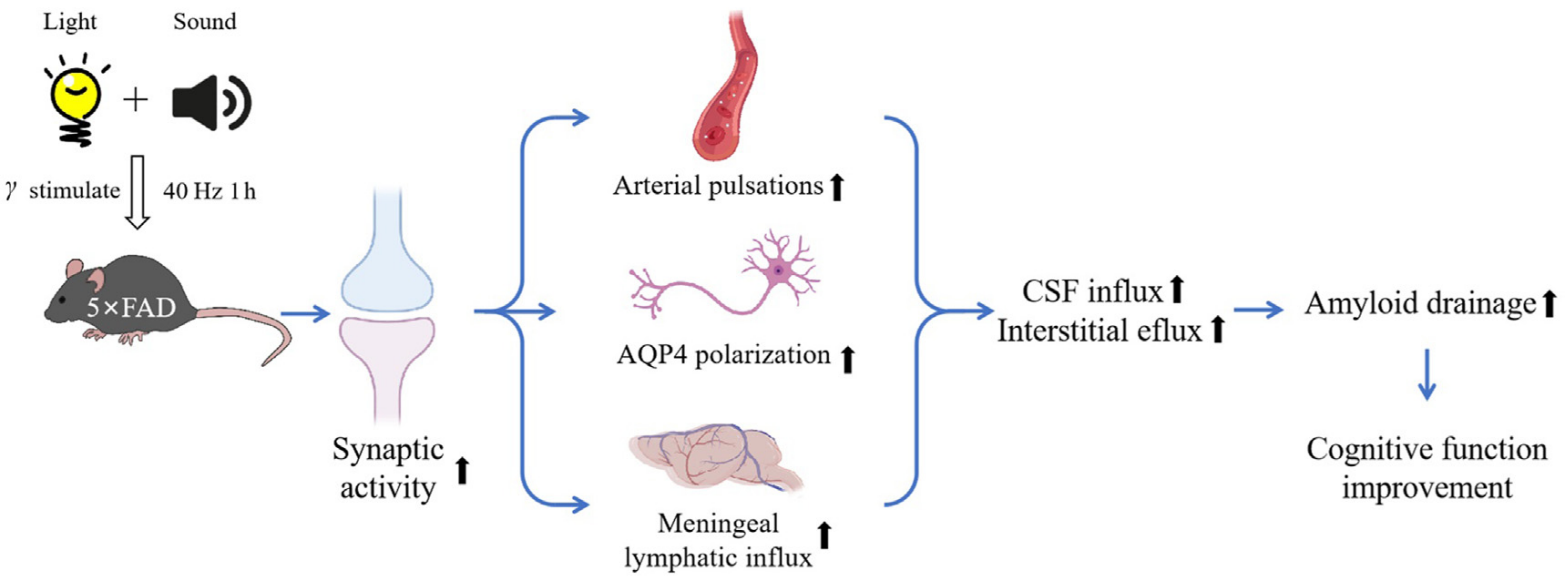
The combination of sound and light stimulation can serve as new approaches for treating neurodegenerative diseases.

Arterial pulsation regulates the movement of CSF^12^. Therefore, the authors wanted to know whether the effect of multisensory 40 Hz stimulation on CSF was due to its influence on pulsation. Arterial vessel imaging in mice revealed that, mice receiving stimulation exhibited an increase in arterial pulsation and an increase in the diameter of lymphatic vessels in the meninges compared to the control group (control mice *n* = 10, *n* = 3 (8 Hz), *n* = 11 (40 Hz), and *n* = 3 (80 Hz) in 6-month-old 5×FAD mice; *P-values* were assessed by one-way ANOVA followed by Dunnett's multiple comparison test). Next, the authors used single-nucleus RNA sequencing (snRNA-seq) to explore the molecular mechanism of CSF influx following gamma stimulation. *Kcnk1* (potassium two pore domain channel subfamily K member 1) gene encodes a highly regulated potassium channel that is thought to be localized to astrocytic endfeet^2^, which are critical for brain fluid transport. The results showed that the puncta signal formed by *Kcnk1* increased significantly with 40 Hz stimulation (*n* = 4 mice per group). The polarity of AQP4 governs glymphatic function and amyloid clearance. Mice receiving multisensory stimulation were observed to have increased polarization of AQP4, suggesting that multisensory 40 Hz stimulation promotes AQP4 polarization. Additionally, RNA sequencing data also showed that VIP (vasoactive intestinal peptide) interneurons release VIP, a 28-amino-acid peptide, during high-frequency stimulation. VIP is not only associated with attenuation of AD pathology but also participates in several cellular processes related to lymphatic clearance, including the regulation of blood vessel diameter, astrocyte metabolism, and aquaporin trafficking. The results proved that the VIP signaling significantly increased in gamma-stimulated mice (*n* = 5 mice without stimulation, *n* = 3 (8 Hz), *n* = 3 (40 Hz) stimulation; *P-values* were calculated by one-way ANOVA followed by Dunnett's multiple comparison test), and chemical genetic inhibition of VIP interneurons before stimulation prevented the increase in arterial pulsation and clearance of A*β*.

The primary benefits of multisensory gamma stimulation lie in its non-invasiveness, high specificity and operational accessibility. By activating the function of the glymphatic system, it promotes the exchange between CSF and ISF, accelerating the clearance of A*β* in the brain, potentially slowing the pathological progression of AD. This finding provides a new perspective in the field of non-pharmacological treatment for AD therapy, emphasizing the crucial role of the glymphatic system in neurodegenerative diseases and providing a new mechanism for A*β* clearance. However, current experiments were only conducted in 6-month-old mice. It remains to be tested whether the multisensory stimulation produces differential effects at multiple stages of AD. Its effects on human still require validation. Future research will need to verify the effects and safety of multimodal gamma stimulation in patients over a broader range of AD stages and at different treatment durations.

## Author contributions

Lixuan Ren drafted the manuscript. Xiwen Ma and Jianping Ye provided the idea and revised the manuscript.

## Conflicts of interest

The authors declare no conflicts of interest.
